# Solitary pulmonary nodule of benign metastasizing leiomyoma associated with primary lung cancer: a case report

**DOI:** 10.1186/1752-1947-5-500

**Published:** 2011-10-05

**Authors:** Masahiro Naito, Tetsu Kobayashi, Masamichi Yoshida, Kentaro Fujiwara, Masahiro Onishi, Atsushi Fujiwara, Takehiro Takagi, Hiroyasu Kobayashi, Esteban C Gabazza, Yoshiyuki Takei, Osamu Taguchi

**Affiliations:** 1 Department of Pulmonary and Critical Care Medicine, Mie University Graduate School of Medicine, Edobashi 2‐174, Tsu, Mie 514‐8507, Japan; 2 Department of Immunology, Mie University Graduate School of Medicine, Edobashi 2‐174, Tsu, Mie 514‐8507, Japan; 3 Department of Gastroenterology and Hepatology, Mie University Graduate School of Medicine, Edobashi 2‐174, Tsu, Mie 514‐8507, Japan; 4 Department of Pulmonary Medicine, Mie Prefectural General Medical Center, Hinaga 5450-132, Yokkaichi, Mie 510-8561, Japan

## Abstract

**Introduction:**

Benign metastasizing leiomyoma in the lung is a very rare disease characterized by the growth of uterine leiomyoma tissue. In most cases there is a previous history of hysterectomy for uterine leiomyoma.

**Case presentation:**

A 50-year-old Asian woman underwent a total abdominal hysterectomy for uterine leiomyoma at the age of 37 years old. She was referred to our hospital because of sudden anterior chest pain. A chest computed tomography scan revealed a ground-glass opacity in her left S10 lung segment and a solitary small nodule in her left bronchial segment, S4. We performed a left lower lobectomy and an upper lung partial resection in order to make a definitive diagnosis and to enable us to determine a further therapeutic strategy. The ground-glass opacity in her left S10 was a primary lung adenocarcinoma, while the small nodule in her left S4 was diagnosed as a benign metastasizing leiomyoma. No additional therapy was done and our patient was followed up with chest computed tomography. Up to date, repetitive evaluation by chest computed tomography has shown no sign of benign metastasizing leiomyoma or lung cancer recurrence.

**Conclusion:**

This is a very rare case of benign metastasizing leiomyoma of the lung associated with primary lung cancer. This comorbid association should be considered in the differential diagnosis when a solitary lung nodule is detected in a patient with a history of uterine leiomyoma.

## Introduction

Benign metastasizing leiomyoma (BML) is a very rare disease characterized by the growth of uterine leiomyoma tissue in the lung [1]. In most cases there is a previous history of hysterectomy for uterine leiomyoma; however, the pathogenesis of the disease has not been as yet elucidated. The comorbid association of primary lung cancer and BML is even more uncommon. Here, we report a case of BML associated with primary lung cancer.

## Case presentation

Our patient was a 50-year-old Asian woman who had undergone a total abdominal hysterectomy for uterine leiomyoma at the age of 37 years old. She did not smoke cigarettes and only drank alcohol socially.

**Figure 1:**
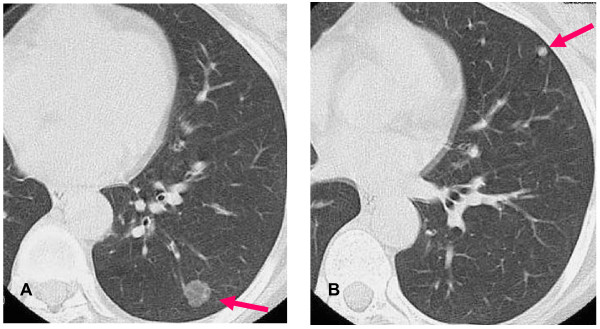
**CT scan of our patient's chest on admission to our hospital**. (A) Chest CT scan shows GGO (arrow) in her left S10 lung segment of 1.3 cm in size. (B) Chest CT scan shows a solitary small nodule (arrow) of 5 mm in diameter in her left S4 segment.

She was referred to our hospital because of sudden anterior chest pain. Hematology, biochemistry and blood gas analysis were normal. A chest computed tomography (CT) scan revealed a ground-glass opacity (GGO) in her left S10 lung segment (Figure 1A) which was 1.3 cm in size, and a solitary small nodule of 5 mm in diameter localized in her left S4 segment (Figure 1B).

**Figure 2:**
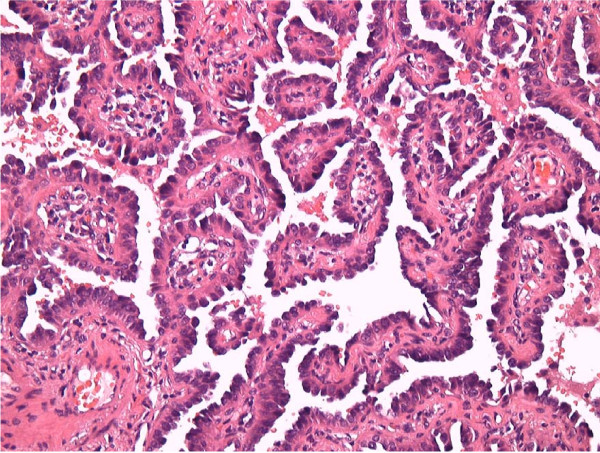
**Histopathology of the lung tumor in her left S10**. Pathological examination of the GGO in her left S10 depicts a localized bronchioloalveolar carcinoma (hematoxylin and eosin staining, × 400).

**Figure 3:**
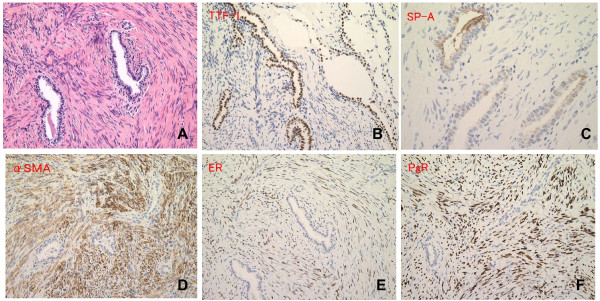
**Histopathology of the lung tumor in her left S4**. (A) Pathological examination of the lung small nodule in her left S4 segment reveals that the tumor is composed of spindle-shaped smooth muscle cells and a low cuboidal metaplastic bronchiolar epithelium, surrounded by fascicles of smooth muscle cells without mitosis or nuclear atypia (hematoxylin and eosin staining, × 400). (B) Immunohistochemical staining for TTF-1 shows positive immunoreactivity in epithelial structures including alveoli or bronchioli (× 400). (C) Immunohistochemical staining for SP-A shows positive immunoreactivity for epithelial structures including alveoli or bronchioli (× 800). (D) Immunohistochemical staining for α-SMA is positive in spindle-shaped cells (× 400). (E) Immunohistochemical staining for ER is positive in spindle-shaped cells (× 400). (F) Immunohistochemical staining for PgR is positive in spindle-shaped cells (× 400).

From the beginning, we suspected lung carcinoma, but we could not get our patient's consent for performing bronchoscopic examination and surgical resection. Follow-up with CT showed that the GGO size had slightly increased and that the small nodule size had not changed. We suspected that the GGO was lung carcinoma, but it was difficult to rule out whether the small nodule was a lung metastasis. If this small nodule was not lung metastasis, the lung carcinoma could have been considered as being in the early stage. We considered that pathological examination by surgical resection was appropriate because it was also an approach for treating the lung carcinoma. We performed a left lower lobectomy and an upper lung partial resection in order to make a definitive diagnosis and to decide further therapeutic strategies. The pathological diagnosis of the GGO in her left S10 segment was primary lung adenocarcinoma(localized bronchioloalveolar carcinoma; Figure 2). On the other hand, pathological examination of the small nodule in her left S4 showed spindle-shaped smooth muscle cells and low cuboidal metaplastic bronchiolar epithelia, surrounded by fascicles of smooth muscle cells without mitosis and nuclear atypia (Figure 3A). Immunohistochemical staining for thyroid transcription factor-1(TTF-1) and surfactant apoprotein A (SP-A) showed epithelial structures composed of alveoli or bronchioli (Figure 3B, C), suggesting that the low cuboidal metaplastic bronchiolar epithelium derived from the pre-existing bronchiolar epithelium. There was positive immunohistochemical staining for α-smooth muscle actin (α-SMA) and spindle-shaped cells (Figure 3D), suggesting that the spindle-shaped cells were smooth muscle cells. Positive immunoreactivity for estrogen receptor (ER) and progesterone receptor (PgR) suggested that the spindle-shaped cells were uterine smooth muscle cells (Figure 3E, F). Unfortunately, a histological sample of the uterine leiomyoma was not available for comparison. The small nodule was diagnosed as a BML based on the results of immunohistochemical staining and her past history of uterine leiomyoma.

No additional therapy was done and follow-up of our patient by chest CT was continued. During four years of follow-up, no recurrence of either the BML or lung cancer could be detected.

## Discussion

BML is a disease in which a tissue from a benign uterine leiomyoma is detected as a solitary nodule or as multiple nodules in the lungs of patients with a previous history of hysterectomy for uterine leiomyoma. In 1939, Steiner *et al*. [1] were the first to report BML as metastasizing fibroleiomyoma of the uterus, and since then there have been several similar reports.

Abramson *et al*. [2] reported that the average age of patients with BML is 48 years old, that the period from hysterectomy to nodule discovery is variable from three months to 26 years and that the first symptom of BML may sometimes be cough or chest pain although it can also be almost indiscernible. Horstmann *et al*. [3] reported that the radiological presentation of BML can be as multiple nodules in 87% of cases (bilateral nodules, 70% and unilateral nodule, 17%) or as a solitary nodule in 13% of cases. The main metastatic site of BML is the lung but other sites, including lymph nodes, soft tissue of the pelvis, bone, bone marrow, greater omentum, peritoneum and heart, have been also reported [4]. Tsunoda *et al*. [5] reported only one case of benign metastasizing leiomyoma of the lung complicated with primary lung cancer. To the best of our knowledge, there are no cases in the literature about the association between lung cancer and BML other than this report. Thus, we believe that this is a very rare case of BML associated with primary lung cancer.

Recent studies have shown that BML is caused by lung metastasis of uterine leiomyoma, which is histologically a benign tumor with a very low grade of malignancy; uterine leiomyoma has been reported to depend on sex hormones [1,6-8]. On the other hand, Patton *et al*. [9] have previously reported that BML results from the monoclonal, hematogenous spread of an apparently benign uterine leiomyoma. However, these conclusions are still controversial.

Pathological examination of the BML in our case showed spindle-shaped cells without mitotic activity or nuclear atypia, surrounded by cuboidal bronchiolar epithelial cells; additional immunohistochemical staining showed that the spindle-shaped cells derived from smooth muscle cells of the uterus, and that the low cuboidal metaplastic cells derived from pre-existing bronchial cells [4]. The presence of TTF-1 is usually assessed to confirm the diagnosis of primary non-small cell lung carcinoma (especially adenocarcinoma) [10]; the purpose of TTF-1 staining in our particular case was to decide whether the low cuboidal metaplastic bronchiolar epithelium observed in the pathological specimens derived from the pre-existing bronchiolar epithelium, because it is known that TTF-1 is only expressed on the normal epithelium of the lung and thyroid [10]. We believe that the diagnosis of BML is not dependent on the expression of TTF-1. Pathological comparison between the solitary pulmonary nodule and the original uterine tumor should provide confirmatory evidence but the sample was not available. However, the small lung nodule was diagnosed as BML based on the results of the immunohistochemical staining and the past history of hysterectomy for uterine leiomyoma.

There is no standard therapy for BML. Recently, Patton *et al*. [9] suggested the possibility of hormonal treatment for BML with positive immunoreactivity for ER and PgR. Other studies have shown improvement of BML after ovariectomy, administration of progesterone or gonadotropin-releasing hormone agonist and menopause [11]. The prognosis of the disease is also unclear. In the present reported case, although the pathological stage of lung carcinoma was stage IA, we considered that CT follow-up was necessary at intervals of three to six months including follow-up of BML recurrence. No additional therapy was done and the follow-up by chest CT showed no recurrence of the BML or lung cancer.

## Conclusion

We report a very rare case of BML associated with primary lung cancer. This comorbid association should be considered in the differential diagnosis when a solitary lung nodule is detected in a patient with a history of uterine leiomyoma.

## Consent

Written informed consent was obtained from the patient for publication of this case report and any accompanying images. A copy of the written consent is available for review by the Editor-in-Chief of this journal.

## Competing interests

The authors declare that they have no competing interests.

## Authors' contributions

NT wrote the manuscript. TK was responsible for the manuscript concept and final corrections to the manuscript. ECG, YT, MY and OT supervised our patient's care and the manuscript. KF, MO, AF, TT and HK participated in patient care as a team. All authors have read and approved the final manuscript.
